# Authorities' Coercive and Legitimate Power: The Impact on Cognitions Underlying Cooperation

**DOI:** 10.3389/fpsyg.2017.00005

**Published:** 2017-01-18

**Authors:** Eva Hofmann, Barbara Hartl, Katharina Gangl, Martina Hartner-Tiefenthaler, Erich Kirchler

**Affiliations:** ^1^Centre for Trust, Peace and Social Relations, Coventry UniversityCoventry, UK; ^2^Faculty of Psychology, University of ViennaVienna, Austria; ^3^Institute of Organization and Global Management Education, Johannes Kepler UniversityLinz, Austria; ^4^Institute of Psychology, Georg-August-University GoettingenGoettingen, Germany; ^5^Labor Science and Organization, Institute of Management Science, TU WienVienna, Austria

**Keywords:** coercive power, legitimate power, trust, authority, cooperation

## Abstract

The execution of coercive and legitimate power by an authority assures cooperation and prohibits free-riding. While coercive power can be comprised of severe punishment and strict monitoring, legitimate power covers expert, and informative procedures. The perception of these powers wielded by authorities stimulates specific cognitions: trust, relational climates, and motives. With four experiments, the single and combined impact of coercive and legitimate power on these processes and on intended cooperation of n_1_ = 120, n_2_ = 130, n_3_ = 368, and n_4_ = 102 student participants is investigated within two exemplary contexts (tax contributions, insurance claims). Findings reveal that coercive power increases an antagonistic climate and enforced compliance, whereas legitimate power increases reason-based trust, a service climate, and voluntary cooperation. Unexpectedly, legitimate power is additionally having a negative effect on an antagonistic climate and a positive effect on enforced compliance; these findings lead to a modification of theoretical assumptions. However, solely reason-based trust, but not climate perceptions and motives, mediates the relationship between power and intended cooperation. Implications for theory and practice are discussed.

## Introduction

In a community, contributions to public goods are often obligatory (e.g., paying taxes in order to finance health services), but some individuals exploit the vulnerable system, refraining from participation, and consequently free ride (Marwell and Ames, 1979). Paying taxes and filing insurance claims are classic real world examples of the free-rider problem. Thus, communities employ regulating formal authorities (e.g., tax administration, insurance companies) with legal measures to persuade free-riders to follow their obligations and to contribute for the benefit of the community. Thereby, we define authorities as processes or individuals which organize the cooperation in a community by an assigned social position that allows to create and maintain environments and thereby influence the behavior of individuals (cf. Andringa et al., 2013). In the current article, we focus on formal authorities. Such authorities have different mechanisms to foster cooperation: the coercive power and the legitimate power (Andreoni et al., 1998; Braithwaite, 2009; Gangl et al., 2013). Employing coercive power, an authority manages behavior with strict monitoring and heavy punishment whereas by using the legitimate power approach, an authority operates through legitimacy of its position, expertise, a policy to disseminate relevant information, and its ability to make others identify with it (Andreoni et al., 1998; Braithwaite, 2009; Gangl et al., 2013, 2015). The slippery slope framework (Kirchler et al., 2008; Gangl et al., 2015) postulates that the perception of both kinds of power stimulate cooperative behavior, but that the underlying cognitions differ.

We shed light on the cognitions that are elicited via coercive and legitimate power of authorities and in turn impact the intention to cooperate. Earlier research shows that coercive power and legitimate power both enhance cooperation in public good dilemmas, where individual interests collide with collective ones (Masclet et al., 2003; Tyler, 2006; Van Lange et al., 2013; Hartl et al., 2015). However, the actual underlying cognitions responsible for the increase in cooperation are not well-understood.

According to the slippery slope framework the perception of authorities' power is assumed to impact individuals' cognitions, such as trust in authorities (implicit and reason-based trust), the relational climate (antagonistic and service climate), and motives for contribution (enforced compliance, voluntary cooperation; Gangl et al., 2015). Implicit trust is diminished when authorities apply coercive power; in contrast, reason-based trust is strengthened by legitimate power. Coercive power induces an antagonistic climate between authorities and individuals. Legitimate power stimulates a service climate. Finally, coercive power leads to enforced compliance, and legitimate power results in voluntarily cooperation.

In this paper, we investigate the cognitions that operate when coercive and legitimate power are wielded to prohibit free-riding (e.g., tax evasion and insurance fraud). The study investigates how coercive power and legitimate power solely or in combination over perceptions of power influence trust in authorities, the climate between authorities and individuals, and the motives of cooperation. Additionally, it analyzes whether the cognitions such as trust, perceived relational climates or motives, mediate the relationship between power and intention to cooperate.

In the remainder of this article, the impact of coercive power and legitimate power on cooperation, trust, relational climates, and motives are defined. Three laboratory experiments in the tax context and one online experiment in the insurance context are described, each assessing the impact of power. Finally, we discuss the results and identify their theoretical and practical implications for legislation and law enforcement.

## The impact of power on cooperation

Power is conceptualized as the capacity of an organization or person to influence another parties' behavior (e.g., Freiberg, 2010; Gangl et al., 2015). Following theory on power (cf. harsh vs. soft power in Raven et al., 1998; coercion vs. persuasion and authority in Turner, 2005; instrumental vs. normative in Tyler et al., 2010) we distinguish between two primary concepts of power, *coercive power* based on deterrence and *legitimate power* based on persuasion (Gangl et al., 2015). Coercive power is defined as “harsh” power, as the capacity to detect and sanction unlawful behavior (Raven et al., 1998; Turner, 2005). Legitimate power is defined as “soft” power and refers to the power of position, expertise, dissemination of relevant information, and identification (Raven et al., 1998, cf. Tyler, 2006). Thus, legitimate power is defined by formal and informal rules established by a rightfully elected government (power of position), and by their knowledge about skillful procedures (power of expertise). In addition, information power and power of identification are seen as means of legitimate power, whereby information, for example, is given on how to behave in accordance with the law, and identification with the authority means that individuals identify with the ideas of the authority such as a specific political party.

Coercive power and legitimate power are two independent forms, which can be wielded exclusively or in combination (cf. French and Raven, 1959; Raven, 1992, 1993; Raven et al., 1998). For instance, wielding coercive power by threatening severe sanctions for unwanted behavior alone is not enough to explain compliant and cooperative behavior (Fehr and Falk, 2002); underlying cognitions such as expectations (Copeland and Cuccia, 2002), reciprocity (Feld and Frey, 2007), and fairness (e.g., Hartner-Tiefenthaler et al., 2012) additionally encourage cooperation. In line with these aspects, we argue that legitimate power, such as distributing information about what the “morally” desired behavior is and the expertly handling of members' contributions to the communal good, becomes important. Empirical evidence shows that coercive power, as well as legitimate power, has a positive impact on cooperative behavior (e.g., Tyler et al., 2010; Hofmann et al., 2014; Hartl et al., 2015). An interaction effect of coercive and legitimate power on cooperation has not been found (e.g., Hofmann et al., 2014; Hartl et al., 2015). Nevertheless, theoretically we would expect that the combination of coercive power and legitimate power is affecting the cognitions underlying cooperative behavior via perception of power (Gangl et al., 2015). Thus, although cooperation might be the same, the underlying cognitions are supposed to be different.

## Power and trust

The application of power is strongly related to trust, whereby trust means “to be vulnerable to the actions of another party based on the expectation that the other will perform a particular action important to the trustor, irrespective of the ability to monitor or control that other party” (Mayer et al., 1995, p. 712). However, the exact nature of the dynamics and relationship between power and trust is not clear. Power was shown to decrease but also to increase trust in authorities (Bachmann, 2001; Bijlsma-Frankema and Costa, 2005; Mulder et al., 2006; Weibel, 2007; Chenhall et al., 2010; Fu et al., 2013). One reason for the divergent results might be that the decision to trust can be either based on reasons or taken implicitly (Castelfranchi and Falcone, 2010), resulting in two forms of trust: implicit trust (system 1 trust) and reason-based trust (system 2 trust). Implicit trust is defined as an automatic and unintentional reaction to stimuli that are associated with positive past experiences or a shared identity. For instance, a taxpayer trusts implicitly in a tax authority, if s/he feels trust immediately without any considerations; this automatic reaction can stem from past positive experiences that ended in a learning process that the tax authority can be trusted. Reason-based trust is defined as a deliberate decision to trust another party based on the evaluation of the other parties' good intentions and internal and external fostering and hindering circumstances to comply with the good intentions (Castelfranchi and Falcone, 2010). Such as a taxpayer weighs whether a tax authority is to be trusted by considering whether the tax authority is pursuing a goal that is valuable to the taxpayer, whether the tax authority is acting motivated, benevolently, and competently, and whether there are external factors fostering or hindering the tax authority's actions.

The slippery slope framework argues that coercive power damages implicit trust (Gangl et al., 2015); as coercion signals authorities' distrust, it may weaken affective and social bonds with authorities, thereby interrupting habitual and implicit cooperation (Kramer, 1999; Das and Teng, 2001). Legitimate power, on the other hand, strengthens trust (Fu et al., 2013); when authorities are perceived as knowledgeable and legitimate in their position, reason-based trust increases. Perceived assistance by experts who work on a transparent legal basis provides many reasons to trust in the competence, motivation, and benevolence of authorities (Bijlsma-Frankema and Van de Bunt, 2002; Malhotra and Murnighan, 2002). For reason-based trust, a strong relationship with legitimate power is assumed because authorities with high levels of legitimate power are perceived as being competent to provide assistance and support (Gangl et al., 2015).

The direct impact of power on trust might in turn also impact cooperation. Thus, trust might be a mediator for the relationship between power and cooperation. However, up to now, most empirical research treats trust as a moderator of the impact of power on cooperation. A meta-analysis shows that power in a trusted environment leads to more cooperation than does power that is exerted in a low-trust environment (Balliet and Van Lange, 2013). Furthermore, experiments show that sanctions exerted by trusted authorities, compared to non-trusted authorities, evoke stronger moral judgments about free-riders (Mulder et al., 2009). There is empirical evidence that power also directly impacts trust (Kramer, 1999; Bijlsma-Frankema and Costa, 2005; Fu et al., 2013). Thus, we assume that trust is not only a moderator but also a mediator between power and cooperation. Coercive and legitimate power impact trust and might consequently influence cooperation with the authorities.

## Power and relational climates

The slippery slope framework postulates that exerting power establishes specific relational climates, whereby climate is defined as the perceived quality of interaction between authorities and individuals (Victor and Cullen, 1988; Martin and Cullen, 2006). This is a “psychological climate that characterizes climate as an individual-level and personal perception” (Ehrhart et al., 2013, p. 70). Two climates can be distinguished in relation to power, the antagonistic climate and the service climate (Kirchler et al., 2008; Gangl et al., 2015). Coercive power and negative experiences with authority trigger an aversive antagonistic climate in which distrust prevails. In such a climate, the authority convicts members of misconduct and suspects others as criminals. In turn, individuals hide from the authority, which justifies stricter controls and sanctions that intensify the vicious circle of distrust (Kirchler et al., 2008).

In contrast, legitimate power and positive impressions of the authorities' intents and work lead to a friendly relational climate in which the authority acts client-oriented. In such a service climate, the authority presents all necessary information for the community members to behave in accordance with the rules. It applies services to support members' cooperation (e.g., preprinted tax forms) to make cooperation easier and non-cooperation more difficult (Gangl et al., 2015).

Empirical research on the impact of power on climates is rare (Alm and Torgler, 2006; Hofmann et al., 2014). Derived from a study on the relationship commitment of business partners (Fu et al., 2013), connections between power and the service climate can be assumed. Legitimate power relates positively to a service climate (i.e., relationship commitment), whereas coercive power relates negatively to it. Based on these results, we predict that in general, coercive power stimulates an antagonistic climate, whereas legitimate power stimulates a service climate. However, the effects on climate when coercive power and legitimate power are exerted simultaneously are not clear as empirical studies are lacking.

## Power and motives for cooperation

Forms of power also encourage different motives for cooperation (Kirchler et al., 2008; Gangl et al., 2015). The punishment aspect of coercive power prompts enforced compliance as threat of severe punishment. Thus, enforced compliance is defined as motive to cooperate because of the deterrent effect of monitoring and punishment (Kirchler et al., 2008). Enforced motivation only leads to cooperation when individuals fear monitoring and punishment and therefore think there is no alternative to comply with the rules (van Meegeren, 2001; Kirchler et al., 2008). Coercive power is effective as long as there are sufficient resources to detect breaches of rules and to undertake subsequent punishment (Becker, 1968; Mulder et al., 2009). In cases in which violations are not discovered or not avenged, coercive power is perceived as weak and, therefore, enforced motives, as well as cooperation decline.

Legitimate power, on the other hand, increases voluntary cooperation. Voluntary cooperation is defined as a motivation to cooperate with the authorities because one wants to reciprocate the positive experience gained through applied legitimate power (Kelman, 2006). Legitimate power activates a felt urge to reciprocate the legitimate treatment (Feld and Frey, 2007). Thus, individuals voluntarily accept their obligation to cooperate. Authorities support customers and clients (e.g., tax authorities offer pre-printed forms that can be submitted without the need for the taxpayer to fill in the form) so that cooperation is perceived as easy and a natural reciprocal act. Although, coercive power and legitimate power are assumed to increase cooperation, the rationale behind cooperation differs fundamentally^1^.

When coercive and legitimate power are applied together, the resulting motives to comply are unclear. Although, results indicate cooperative behavior based on coercive and legitimate power, the underlying cognitions are still unexplored (Hofmann et al., 2014; Hartl et al., 2015). First, empirical evidence indicates that people cooperate voluntarily when legitimate power is high, but only under the condition that rule-breakers can be punished (Kroll et al., 2007). Thus, the combination of coercive and legitimate power seems to increase voluntary cooperation and enforced compliance. In general we assume that the combination of coercive power and legitimate has the same impact as if coercive power and legitimate power were applied solely.

## Overview of studies

We examine the cognitions underlying the intentions to cooperate in different social dilemma situations. In order to investigate differences in cognitions induced by extremely low or high levels of coercive and/or extremely low or high levels of legitimate power an experimental design is opted for. The experiments allow for controlling other possible influences and showing the pure influence of coercive and legitimate power.

The current studies were embedded in a broader research program testing the impact of the two forms of power—solely and combined. Hartl et al. (2015) examined the impact of beliefs of coercive and legitimate power on tax behavior and found a significant effect of both on experimental cooperative behavior. However, so far, the underlying and probably mediating cognitions of why people intent to cooperate with authority have not been analyzed. Hence, this study investigates the underlying cognitions of this behavior. As such, we analyze intended tax compliance but not behavior (partial correlation controlling for conditions between tax honesty intention and tax payments is *r* = 0.58, *p* < 0.001 in Study 1, *r* = 0.60, *p* < 0.001 in Study 2, and *r* = 0.64, *p* < 0.001 in Study 3). We examine the following three hypotheses:

Hypothesis 1a: *Coercive power leads to low levels of implicit trust, an antagonistic climate, and enforced compliance*.
Hypothesis 1b: *Coercive power leads to low levels of implicit trust, an antagonistic climate, and enforced compliance, when at the same time legitimate power is wielded*.
Hypothesis 2a: *Legitimate power leads to reason-based trust, a service climate, and voluntary cooperation*.
Hypothesis 2b: *Legitimate power leads to reason-based trust, a service climate, and voluntary cooperation, when at the same time coercive power is wielded*.
Hypothesis 3: *The relationship between coercive power and/or legitimate power and intended cooperation is mediated by implicit trust, reason-based trust, the antagonistic climate, the service climate, enforced compliance and voluntary cooperation*.

To test these hypotheses, following standard procedures (cf. Kirchler et al., 2009) we conducted laboratory experiments and an online experiment. In the laboratory experiments, participants imagined being a taxpayer in a fictitious country (Chomland) in which tax authorities wield coercive power (Study 1) or legitimate power (Study 2) exclusively or in combination (Study 3). In the online experiment, coercive and legitimate power were manipulated in combination, but rather than investigating tax compliance, the decision concerned an insurance claim (Study 4).

To measure the level of cooperation, participants had to decide how much of their income they declare honestly to pay taxes and how much they claim at the insurance for compensation, respectively. For reasons of comparison, the designs of the four studies and the procedures are similar, facilitating conclusions on the effects of the different forms of power across various contexts. A between-subjects design of the laboratory experiments assured that participants were confronted with low or high forms of power.

## Study 1: coercive power in the tax context

### Methods

#### Participants

In all, 120 students (64% men, *M*_age_ = 24.48, *SD* = 5.85) majoring in several different disciplines participated on a voluntary basis and were paid according to their behavior in the experiment. As student populations are specifically naïve regarding experiences with tax authorities and our hypotheses, they specifically suit hypotheses testing in this context (Mittone, 2006).

#### Experimental design and procedure

The study was conducted by randomly assigning participants to one of two conditions. All participants were asked to imagine being self-employed taxpayers in the fictitious country Chomland with a hypothetical tax authority (for similar tax experiments see, e.g., Kirchler et al., 2009; Andrighetto et al., 2016). Specifically participants learnt “In each period a certain income is allocated to you, of which you have to pay taxes. The tax rate is 40% of your income. In each period your final income is the result of the allocated income minus the taxes paid. At the end of the experiment one period will be selected randomly. The income that you have gained in this period will be paid to you by the experimenter. Additionally, for each period there exists a tax audit probability of 15%. In case you are audited and you have evaded taxes, you have to pay back the evaded amount plus a fine of 1 time the evaded amount.” The final income was paid out by the experimenter.

In the two conditions, the tax authority held either low or high levels of coercive power. A tax authority with low/high levels of coercive power was, for example, described as “… well-known for its lenient/hard sanctions.” After participants were introduced to the experimental set-up, a taxpayer's life was simulated using the software z-Tree (Fischbacher, 2007). Participants were asked to answer two items about their intention to pay taxes honestly in this situation (tax honesty intention, two items; e.g., “How likely is it that you, as a citizen of Chomland, state your income and expenses totally correctly?”). After that, participants paid their taxes, whereby at the end of the experiment participants were remunerated based on their behavior [participants received on average 5.99 EUR (*SD* = 1.22) or 7.66 USD (*SD* = 1.56), respectively].

#### Material

In all treatment conditions, participants had to fill out a questionnaire. The questionnaire assessed implicit trust (three items; e.g., “I trust the tax authority in Chomland without thinking about it.”), reason-based trust (seven items; e.g., “I trust the tax authority in Chomland because it gives me competent advice.”), the antagonistic climate (three items; e.g., “Between the tax authority in Chomland and taxpayers there exists a climate of ruthlessness.”), the service climate (three items; e.g., “Between the tax authority in Chomland and taxpayers there exists a climate that is characterized by its service-oriented nature.”), enforced compliance (three items; e.g., “When I pay taxes according to the law in Chomland, I do so because the tax authority often carries out audits.”), and voluntary cooperation (three items; e.g., “When I pay taxes according to the law in Chomland, I do so because the tax authority supports taxpayers who make unintentional mistakes.”). As a manipulation check, we asked participants' perceptions of the tax authority's coercive power (four items; e.g., “I believe that the tax authority persecutes taxpayers with audits and fines.”) and legitimate power (22 items; e.g., “I believe that the tax authority knows how to give good advice to taxpayers.”) by adapting published scales from the organizational context (Hinkin and Schriesheim, 1989; Raven et al., 1998) to the tax context (all items are listed in Supplementary Material). The scale of legitimate power compounded four sub-scales (legitimacy, expertise, information, identification), but for the sake of simplicity and due to the tested measurement models, the sub-scales were combined into one scale. Responses were indicated on a seven-point Likert scale ranging from 1 (“*I totally disagree*”) to 7 (“*I totally agree*”). Cronbach's α for the eight scales were excellent and can be found in Table 1.

**Table 1:** **Study 1: Results of the ANOVAs with *coercive power* as independent variable**.

**Dependent variables**	**α**	***F* (df_1_, df_2_)**	***p***	**η_*p*_^2^**
Perceptions of coercive power	0.93	139.26 (1, 118)	<0.001	0.54
Perceptions of legitimate power	0.90	0.37 (1, 118)	0.55	<0.01
Implicit trust	0.89	3.27 (1, 118)	0.07	0.03
Reason-based trust	0.84	0.00 (1, 118)	1.00	<0.01
Antagonistic climate	0.78	9.80 (1, 118)	<0.01	0.08
Service climate	0.76	1.00 (1, 118)	0.32	0.01
Enforced compliance	0.94	57.97 (1, 118)	<0.001	0.33
Voluntary cooperation	0.74	0.03 (1, 118)	0.87	<0.01
Intended tax honesty	0.90	6.61 (1, 118)	<0.05	0.05

*α, Cronbach α*.

Socio-demographics (gender, age, income, nationality, employment, form of employment, working hours, and experience with tax authorities) were also assessed.

### Results

#### Preliminary data analyses

To check whether the manipulation of coercive power was successful, an ANOVA^2^ was performed with the perceptions of coercive power as the dependent variable. The results showed that the manipulation was successful as low (cp_low_) and high (cp_high_) levels of perceptions regarding coercive power were in line with the manipulation (cp_low_: *M* = 2.60, *SD* = 1.34; cp_high_: *M* = 5.51, *SD* = 1.37; Table 1). The manipulation of coercive power had no significant effect on the perceptions of legitimate power (Table 1). Participants experiencing low or high levels of coercive power reported equal perceptions of legitimate power (cp_low_: *M* = 4.13, *SD* = 1.04; cp_high_: *M* = 4.24, *SD* = 0.90; see Table 1).

#### Coercive power

##### The impact of coercive power on trust, climates, and motives

**Figure 1:**
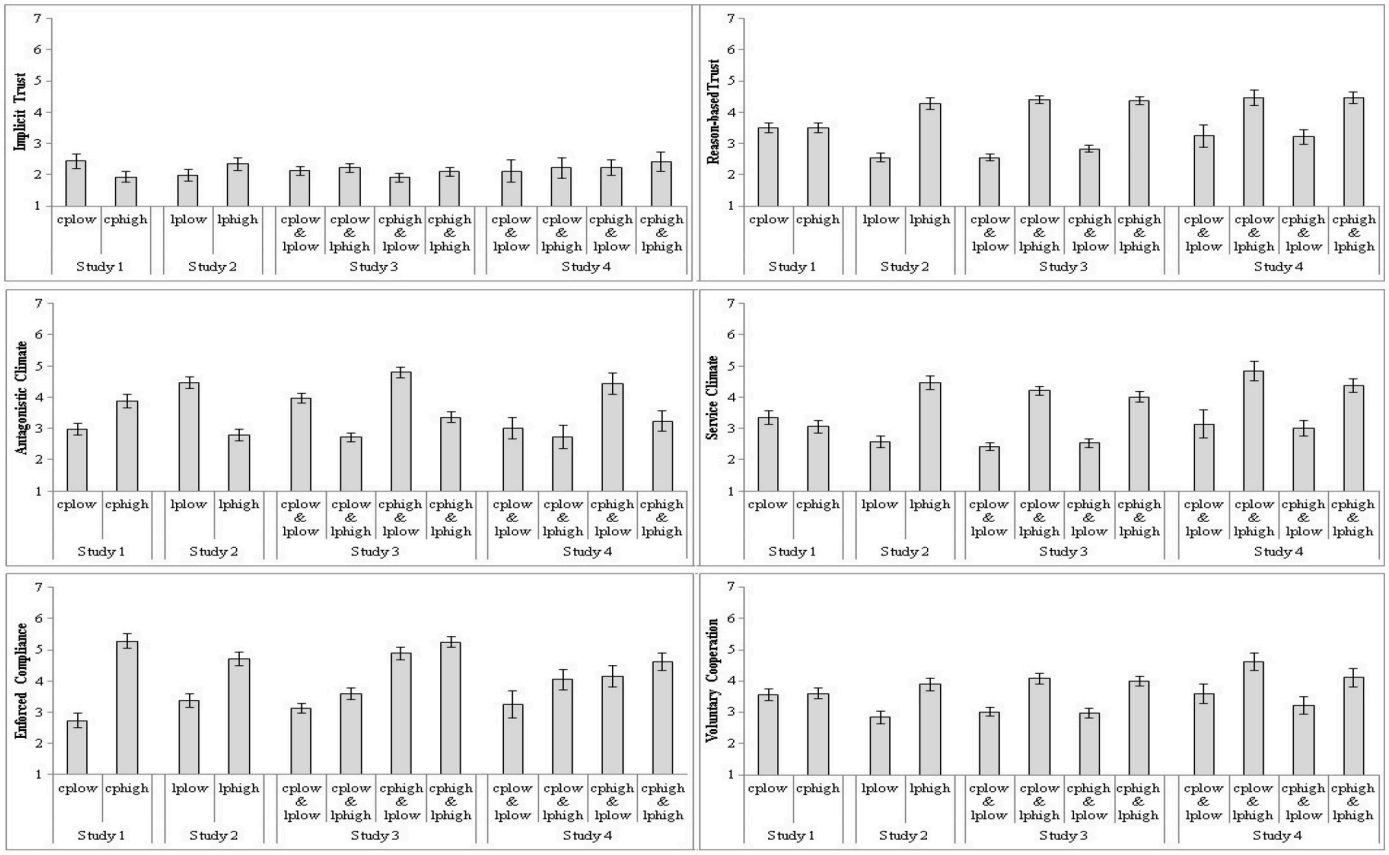
**The impact of coercive and legitimate power on the trust, climate, and motive scales in Study 1–4**.

To test Hypothesis 1a, ANOVAs were conducted, including coercive power (low vs. high) as factor variables and implicit and reason-based trust, antagonistic and service climate, and enforced compliance and voluntary cooperation as dependent variables (see Table 1 for ANOVA results; for a graphical representation see Figure 1 in the Discussion Section). As expected, coercive power showed a tendency to decrease implicit trust (cp_low_: *M* = 2.43, *SD* = 1.70; cp_high_: *M* = 1.93, *SD* = 1.31; Table 1). Furthermore, no matter whether participants experienced low or high levels of coercive power, they reported an equal intensity of reason-based trust (cp_low_: *M* = 3.50, *SD* = 1.28; cp_high_: *M* = 3.50, *SD* = 1.25; Table 1).

Regarding the perception of the relational climate, the analysis showed that, as expected, high levels of coercive power led to a higher perception of an *antagonistic climate* (cp_high_: *M* = 3.86, *SD* = 1.68) compared to when coercive power was low (cp_low_: *M* = 2.96, *SD* = 1.47; Table 1). *Service climate* was not affected by different levels of coercive power (cp_low_: *M* = 3.34, *SD* = 1.61; cp_high_: *M* = 3.05, *SD* = 1.55; Table 1).

Regarding the motives for cooperation, as expected, participants felt more *enforced* to comply when coercive power was high (cp_high_: *M* = 5.27, *SD* = 1.80) rather than low (cp_low_: *M* = 2.73, *SD* = 1.86; Table 1). Participants experiencing low or high levels of coercive power reported equal levels of *voluntary cooperation* (cp_low_: *M* = 3.56, *SD* = 1.51; cp_high_: *M* = 3.61, *SD* = 1.42; see Table 1).

##### The mediating role of implicit trust, the antagonistic climate, and enforced compliance

Investigating Hypothesis 3 regarding whether implicit trust, the perception of the antagonistic climate and enforced compliance mediate the relationship between coercive power and the intention to pay taxes honestly, we first tested, using an ANOVA, whether or not the manipulation of coercive power impacted the intention to pay taxes honestly. The manipulation of high levels of coercive power lead to higher tax honesty intention (*M* = 4.92, *SD* = 1.70) compared to low levels of coercive power (*M* = 4.10, *SD* = 1.78; Table 1).

In a second step, we applied the program Mediate (Hayes et al., 2011) to test whether the relationship between coercive power and tax honesty intention is mediated by the proposed variables (i.e., implicit trust, antagonistic climate, and enforced compliance) at the same time. With this method, we received outcomes on simple (mediators and criterion regressing on predictor) and multivariate linear regressions (criterion regressing on mediators and on predictor; Hayes et al., 2011; Hayes, 2013).

The mediator analysis revealed one indirect effect from coercive power to tax honesty intention, the relation was only found to be mediated by implicit trust (95% CI [−0.31; −0.01]; Table 2). However, Sobel test statistics (Sobel test = −1.51, *p* = 0.13) do not indicate a significant mediation.

**Table 2:** **Study 1: Mediation analysis from coercive power to tax honesty intention (THI) (standard errors in parentheses)**.

	**Path coefficients**				**Indirect effects**		
	**To THI**	**To IT**	**To AC**	**To EC**	**Estimate**	**Sobel Z**	**Symmetric 95% CI**
From coercive power (CP)	0.77 (0.38)	−0.50 (0.28)	0.90 (0.29)	2.54 (0.33)			
From implicit trust (IT)	0.28 (0.10)						
From antagonistic climate (AC)	−0.03 (0.11)						
From enforced compliance (EC)	0.08 (0.90)						
CP→IT→THI					−0.14 (0.10)	−1.505	−0.31; −0.01
CP→AC→THI					−0.03 (0.10)	−0.271	−0.20; 0.13
CP→EC→THI					0.21 (0.24)	0.088	−0.17; 0.61

### Discussion

As predicted, coercive power generally has a negative impact on implicit trust and initiates the perception of an antagonistic climate and enforced compliance, overall confirming hypothesis 1a. Coercive power applied alone does not impact reason-based trust, the perception of a service climate or voluntary cooperation. In addition, the relationship between coercive power and intended tax honesty seems not to be mediated. Implicit trust, a perceived antagonistic climate and the enforced motive to cooperate are not mediators. Thus, Hypothesis 3 is not confirmed.

## Study 2: legitimate power in the tax context

### Methods

#### Participants

Overall, 130 students (60% men, *M*_age_ = 24.40, *SD* = 4.86) majoring in different fields participated on a voluntary basis and were paid based on their behavior in the experiment. Again, this population was selected because of their naivety regarding experiences with tax authorities (Mittone, 2006).

#### Experimental design and procedure

The experimental design and procedure was similar to Study 1. Two conditions were used, in which the tax authority was described as holding either low or high levels of legitimate power. The tax authority with low levels of legitimate power was characterized as being, for example, “poorly appreciated for its work”; the ones holding high levels of legitimate power were presented as, for example, being “highly appreciated for its work.” The scenario contains all aspects of legitimate power (legitimacy, expertise, dissemination of information, and identification). Cronbach's α for the eight scales were excellent and can be found in Table 3. The participants were remunerated according to their behavior and received, on average, 6.40 EUR (*SD* = 1.38) or 8.18 USD (*SD* = 1.76), respectively.

**Table 3:** **Study 2: Results of the ANOVAs with *legitimate power* as independent variable**.

**Dependent variables**	**α**	***F* (df_1_, df_2_)**	***p***	**η_*p*_^2^**
Perceptions of coercive power	0.89	13.38 (1, 128)	<0.001	0.10
Perceptions of legitimate power	0.95	79.66 (1, 128)	<0.001	0.38
Implicit trust	0.88	1.84 (1, 128)	0.18	0.01
Reason-based trust	0.89	59.04 (1, 128)	<0.001	0.32
Antagonistic climate	0.83	41.15 (1, 128)	<0.001	0.24
Service climate	0.88	47.11 (1, 128)	<0.001	0.27
Enforced compliance	0.91	19.75 (1, 128)	<0.001	0.13
Voluntary cooperation	0.85	13.81 (1, 128)	<0.001	0.10
Intended tax honesty	0.78	20.38 (1, 128)	<0.001	0.14

*α, Cronbach α*.

### Results

#### Preliminary data analyses

The manipulation was successful as low and high levels of legitimate power induced perceptions according to the manipulation (lp_low_: *M* = 3.16, *SD* = 1.05; lp_high_: *M* = 4.89, *SD* = 1.16; Table 2). Surprisingly, the analysis showed that the manipulation of legitimate power had a significant impact on the perceptions of coercive power (Table 3). The perceptions of coercive power were higher when legitimate power was high (lp_high_: *M* = 4.51, *SD* = 1.57) rather than low (lp_low_: *M* = 3.48, *SD* = 1.64).

#### Legitimate power

##### The impact of legitimate power on trust, climates, and motives.

Testing Hypothesis 2a, ANOVAs were conducted including legitimate power (low vs. high) as factor and implicit and reason-based trust, antagonistic and service climate and enforced compliance and voluntary cooperation as dependent variables (see Table 3 for ANOVA results; for a graphical representation see Figure 1 in the Discussion Section). Regardless of whether or not participants experienced low or high levels of legitimate power, they reported an equal intensity of implicit trust (lp_low_: *M* = 1.97, *SD* = 1.55; lp_high_: *M* = 2.34, *SD* = 1.54; see Table 3). As expected, participants experiencing high levels of legitimate power reported high levels of reason-based trust (lp_low_: *M* = 2.55, *SD* = 1.09; lp_high_: *M* = 4.29, *SD* = 1.48; see Table 3).

Regarding the perception of the relational climate, unexpectedly the analysis revealed that low levels of legitimate power led to a higher perception of an *antagonistic climate* (lp_low_: *M* = 4.47, *SD* = 1.47) compared to when legitimate power was high (lp_high_: *M* = 2.80, *SD* = 1.48). In line with the hypothesis, the perception of a *service climate* increased with legitimate power (lp_low_: *M* = 2.57, *SD* = 1.50; lp_high_: *M* = 4.46, *SD* = 1.64; see Table 3).

Regarding the motives for cooperation, participants in the condition of high levels of legitimate power felt more *enforced* to comply (lp_high_: *M* = 4.70, *SD* = 1.68) compared to participants in the condition of low levels of legitimate power (lp_low_: *M* = 3.36, *SD* = 1.75). In line with predictions, participants experiencing high levels of legitimate power reported higher levels of *voluntary cooperation* (lp_high_: *M* = 3.90, *SD* = 1.61) than did participants experiencing low levels of legitimate power (lp_low_: *M* = 2.84, *SD* = 1.65; see Table 3).

##### The mediating role of reason-based trust, the service climate, and voluntary cooperation.

Testing Hypothesis 3, we first investigated for the impact of legitimate power on tax honesty intention. The ANOVA revealed that high levels of legitimate power lead to higher tax honesty intention (*M* = 4.92, *SD* = 1.43) compared to low levels of legitimate power (*M* = 3.74, *SD* = 1.55; Table 3).

In a second step, we used Mediate (Hayes et al., 2011) for the mediator analysis. The findings showed that an indirect effect from legitimate power to tax honesty intention was solely explained by reason-based trust (95% CI [0.05; 0.94]; Table 4). Also Sobel test statistics (Sobel test = 1.81, *p* = 0.07; *R*_M_ = 0.62^3^) do by trend indicate this significant mediation.

**Table 4:** **Study 2: Mediation analysis from legitimate power to tax honesty intention (THI) (standard errors in parentheses)**.

	**Path coefficients**				**Indirect effects**		
	**To THI**	**To RBT**	**To SC**	**To VC**	**Estimate**	**Sobel Z**	**Symmetric 95% CI**
From legitimate power (LP)	0.79 (0.32)	1.74 (0.23)	1.89 (0.28)	1.06 (0.29)			
From reason-based trust (RBT)	0.28 (0.15)						
From service climate (SC)	−0.10 (0.11)						
From voluntary cooperation (VC)	0.09 (0.11)						
LP→RBT→THI					0.48 (0.27)	1.812	0.05; 0.94
LP→SC→THI					−0.18 (0.20)	0.900	−0.51; 0.15
LP→VC→THI					0.09 (0.12)	0.798	−0.09; 0.30

### Discussion

Consistent with Hypothesis 2a, high levels of legitimate power have a positive effect on reason-based trust, on the perception of a service climate and on voluntary cooperation. Not hypothesized, high levels of legitimate power also increase perceptions of coercive power, and higher enforced compliance. Furthermore, legitimate power profoundly reduces the perception of an antagonistic climate. Although, coercive power was assumed to be the only quality of power to have an impact on enforced compliance and the perception of an antagonistic climate, the findings point out that legitimate power is additionally interfering. Regarding Hypothesis 3, only reason-based trust is by trend mediating the relationship between legitimate power and tax honesty intention. In the third experiment, the relationship between coercive power and legitimate power is examined.

## Study 3: coercive power and legitimate power combined in the tax context

### Methods

#### Participants

Analogous to Study 1 and 2, 368 students (34% men, *M*_age_ = 24.26, *SD* = 5.56) majoring in different disciplines participated in the experiment and were paid based on their behavior in the experiment.

#### Experimental design and procedure

Experimental design and procedure were similar to Studies 1 and 2, but four conditions were designed in which the hypothetical tax authority held either low or high levels of coercive power *and* low or high levels of legitimate power. The combination of low/high levels of coercive power and *low/high levels of legitimate power* was operationalized through scenarios (e.g., “In general, the tax authority is known for its low/high penalties for tax evasion, and is *little/very* appreciated for its work.”). Cronbach's α's are excellent and are presented in Table 3. Participants were remunerated according to their behavior and received, on average, 6.21 EUR (*SD* = 1.32) or 7.94 USD (*SD* = 1.69), respectively.

### Results

In the following, only hypothesized and/or significant results are reported; however, for completeness, Table 5 displays all findings independently of whether or not they were significant.

**Table 5:** **Study 3: Results of the ANOVAs with *coercive power* and *legitimate power* as independent variables**.

**Dependent variables**		**α**	***F* (df_1_, df_2_)**	***p***	**η_*p*_^2^**
Perceptions of coercive power		0.91			
	CP		459.61 (1, 364)	<0.001	0.56
	LP		5.71 (1, 364)	0.02	0.02
	CPxLP		0.18 (1, 364)	0.68	<0.01
Perceptions of legitimate power		0.94			
	CP		0.26 (1, 364)	0.61	<0.01
	LP		260.33 (1, 364)	<0.001	0.42
	CPxLP		0.07 (1, 364)	0.79	<0.01
Implicit trust		0.86			
	CP		1.42 (1, 364)	0.24	<0.01
	LP		0.94 (1, 364)	0.33	<0.01
	CPxLP		0.09 (1, 364)	0.76	<0.01
Reason-based trust		0.86			
	CP		1.29 (1, 364)	0.26	<0.01
	LP		217.19 (1, 364)	<0.001	0.37
	CPxLP		1.78 (1, 364)	0.18	<0.01
Antagonistic climate		0.84			
	CP		21.40 (1, 364)	<0.001	0.06
	LP		70.871 (1, 364)	<0.001	0.16
	CPxLP		0.33 (1, 364)	0.56	<0.01
Service climate		0.85			
	CP		0.09 (1, 364)	0.76	<0.01
	LP		128.81 (1, 364)	<0.001	0.26
	CPxLP		1.08 (1, 364)	0.30	<0.01
Enforced compliance		0.92			
	CP		90.24 (1, 364)	<0.001	0.20
	LP		5.49 (1, 364)	0.02	0.02
	CPxLP		0.07 (1, 364)	0.79	<0.01
Voluntary cooperation		0.83			
	CP		0.11 (1, 364)	0.75	<0.01
	LP		45.37 (1, 364)	<0.001	0.11
	CPxLP		0.02 (1, 364)	0.89	<0.01
Intended tax honesty		0.86			
	CP		34.50 (1, 364)	<0.001	0.09
	LP		19.75 (1, 364)	<0.001	0.05
	CPxLP		0.04 (1, 364)	0.85	<0.01

*α, Cronbach α; CP, main effect coercive power; LP, main effect legitimate power; CPxLP, interaction effect of coercive and legitimate power*.

#### Preliminary data analyses

Checking the coercive power manipulation with the participants' perceptions of coercive power, the ANOVA showed that low and high levels of coercive power conditions induced respective perceptions (cp_low_: *M* = 2.67, *SD* = 1.21; cp_high_: *M* = 5.50, *SD* = 1.33; Table 5).

Likewise, a manipulation check for legitimate power confirmed the manipulation (Table 5). Participants experiencing high levels of legitimate power reported perceptions of higher legitimate power (lp_high_: *M* = 4.82, *SD* = 0.94) than did participants who experienced low levels of legitimate power (lp_low_: *M* = 3.20, *SD* = 0.99). Similar to Study 2, the analysis showed that the manipulation of legitimate power had a significant impact on the perceptions of coercive power (Table 5). Participants perceived coercive power to be stronger when legitimate power was high (lp_high_: *M* = 4.82, *SD* = 0.94) rather than low (lp_low_: *M* = 3.20, *SD* = 0.99).

#### Coercive power and legitimate power

##### The impact of coercive and legitimate power on trust, climates, and motives

Testing Hypotheses 1b and 2b, 2 (low vs. high levels of coercive power) by 2 (low vs. high levels of legitimate power) ANOVAs with the depending variables implicit and reason-based trust, antagonistic and service climate and enforced compliance and voluntary cooperation were applied. Contrary to expectations, participants in conditions with low/high levels of coercive power and low/high levels of legitimate power reported equal intensity of *implicit trust* (main effects: cp_low_: *M* = 2.17, *SD* = 1.39; cp_high_: *M* = 2.00, *SD* = 1.33; lp_low_: *M* = 2.02, *SD* = 1.39; lp_high_ *M* = 2.16, *SD* = 1.33; see Table 5; for a graphical representation see Figure 1 in the Discussion Section). Regarding *reason-based trust*, the analysis revealed, as expected, that participants reported higher levels of reason-based trust when *legitimate power* was high (lp_high_: *M* = 4.39, *SD* = 1.17; lp_low_: *M* = 2.69, *SD* = 1.04; see Table 5).

Regarding the perception of the relational climate, the analysis showed that the perception of an *antagonistic climate* increased with coercive power (cp_low_: *M* = 3.35, *SD* = 1.51; cp_high_: *M* = 4.08, *SD* = 1.81) and decreased with legitimate power (lp_low_: *M* = 3.38, *SD* = 1.58; lp_high_: *M* = 3.03, *SD* = 1.56; see Table 5). Furthermore, as expected, the analysis showed that the perception of a *service climate* increased with legitimate power (lp_low_: *M* = 2.47, *SD* = 1.33; lp_high_: *M* = 4.11, *SD* = 1.43, see Table 5).

Regarding the motives for cooperation, the analysis highlighted that, as expected, in conditions with low levels of coercive power, participants reported feeling less *enforced* than in conditions with high levels of coercive power (cp_low_: *M* = 3.35, *SD* = 1.67; cp_high_: *M* = 5.06, *SD* = 1.80; see Table 5). Furthermore, as expected, participants reported more *voluntary cooperation* when legitimate power was high (lp_high_: *M* = 4.04, *SD* = 1.55; lp_low_: *M* = 2.99, *SD* = 1.43, see Table 5). No other main effects and no interaction effects were significant (see Table 5).

##### The mediating role of trust, climate, and motive.

Testing Hypothesis 3, the ANOVA found that coercive and legitimate power had a significant impact on intended tax honesty and that no significant interaction existed. High levels of coercive power led to higher tax honesty intention (*M* = 4.98, *SD* = 1.41) than did low levels of coercive power (*M* = 4.02, *SD* = 1.78). Similarly, manipulations of high levels of legitimate power stimulated higher tax honesty intention (*M* = 4.86, *SD* = 1.50) than did lower legitimate power (*M* = 4.13, *SD* = 1.76; Table 5).

In a second step, we again used Mediate (Hayes et al., 2011) for the mediator analysis, this time working with two predictors, i.e., coercive power and legitimate power. The results showed that there is only one indirect effect, that is, from legitimate power to tax honesty intention via reason-based trust (95% CI [0.10; 0.65]; Table 6). Also Sobel test statistics (Sobel test = 2.11, *p* = 0.03; *R*_M_ = 0.37) do indicate this significant mediation. All the other indirect effects from coercive and legitimate power are not significant.

**Table 6:** **Study 3: Mediation analysis from power to tax honesty intention (THI; standard errors in parentheses)**.

	**Path coefficients**							**Indirect effects**		
	**To THI**	**To IT**	**To RBT**	**To AC**	**To SC**	**To EC**	**To VC**	**Estimate**	**Sobel Z**	**Symmetric 95% CI**
From covercive power (CP)	1.02 (0.18)	−0.17 (0.14)	0.13 (0.12)	0.74 (1.16)	−0.04 (0.14)	1.71 (0.18)	−0.05 (0.16)			
From legitimate power (LP)	0.32 (0.22)	0.14 (0.14)	1.71 (0.12)	−1.34 (0.16)	1.64 (0.14)	0.42 (0.18)	1.05 (0.16)			
From implicit trust (IT)	0.04 (0.06)									
From reason-based trust (RBT)	0.22 (0.10)									
From antagonistic climate (AC)	−0.04 (0.06)									
From service climate (SC)	−0.05 (0.07)									
From enforced compliance (EC)	−0.03 (0.05)									
From voluntary cooperation (VC)	0.08 (0.07)									
CP→IT→THI								−0.01 (0.02)	−0.584	−0.04; 0.01
LP→IT→THI								0.01 (0.01)	0.554	−0.01; 0.03
CP→RBT→THI								0.03 (0.03)	0.972	−0.01; 0.08
LP→RBT→THI								0.37 (0.17)	2.174	0.10; 0.65
CP→AC→THI								−0.03 (0.05)	−0.461	−0.10; 0.04
LP→AC→THI								0.05 (0.08)	0.664	−0.08; 0.18
CP→SC→THI								0.00 (0.01)	0.265	−0.02; 0.02
LP→SC→THI								−0.09 (0.11)	−0.831	−0.28; 0.10
CP→EC→THI								−0.05 (0.08)	−0.599	−0.19; 0.09
LP→EC→THI								0.09 (0.08)	−0.581	−0.05; 0.02
CP→VC→THI								−0.00 (0.02)	−0.301	−0.04; 0.02
LP→VC→THI								0.09 (0.08)	1.126	−0.04; 0.21

### Discussion

The analyses partly confirm Hypotheses 1b and 2b. The manipulation of coercive power and legitimate power at the same time in the context of taxpaying confirmed that in cases of high levels of coercive power, the antagonistic climate and enforced compliance are more distinct. In addition, higher legitimate power induced reason-based trust, a distinct service climate and voluntary cooperation. Contrary to Hypothesis 1b, high levels of coercive power did not reduce implicit trust. Also, high levels of legitimate power fundamentally reduced an antagonistic climate. Regarding the mediating effect testing Hypothesis 3, only reason-based trust mediated the relationship between legitimate power and tax honesty intention. In Study 4 the impact of coercive power and legitimate power is investigated in another situation.

## Study 4: coercive power and legitimate power combined in the insurance context

### Methods

#### Participants

Overall, 102 students (83% men, *M*_age_ = 22.66, *SD* = 3.12) majoring in industrial engineering participated in the study. For participation, all students received bonus points for one of their courses. Again, this population primarily was selected because of their naivety regarding the hypotheses and because of their inexperience with insurance organizations.

#### Experimental design and procedure

In contrast to Studies 1–3, in Study 4 scenarios in an online experiment were used in which an insurance organization was presented as wielding high or low levels of coercive power and high or low levels of legitimate power. Participants were randomly assigned to one of four conditions. The combination of low/high levels of coercive power and of low/high levels of *legitimate power* was operationalized through items such as, “In general, the insurance company is known for its low/high penalties for insurance fraud. It is *little/very appreciated* for its work.” After the scenarios, the respondents had to report damage to the insurance organization. They had to “… imagine that [their] television set broke from the wall so that it was now in pieces. The television set had a value of 600 MU [MU, monetary units], which [they] had to report to the insurance company according to the terms. With the help of a friend, who can fake an invoice up to a maximum of 1000 MU, [they] could report a higher claim to the insurance company.” The amounts of respondents' claims (ranging from 600 to 1.000 MU) were collected to assess their relative cooperation with the insurance organization and will further be displayed in percentages [(reported amount − 600)/400]. The questionnaire that was used in Studies 1–3 was adapted to the insurance context and was applied to measure insurance fraud intention [one item; “Which damage sum would you claim at the insurance company (min. 600 MU, max. 1000 MU):”], implicit trust (three items; e.g., “I trust the insurance company Chom-Insurance without thinking about it.”), reason-based trust (seven items; e.g., “I trust the insurance company Chom-Insurance, because it gives me competent advice.”), the antagonistic climate (three items; e.g., “Between the insurance company Chom-Insurance and the insurants, there exists a climate of ruthlessness.”), the service climate (three items; e.g., “Between the insurance company Chom-Insurance and the insurants, there exists a climate, which is characterized by its service-oriented nature.”), enforced compliance (three items; e.g., “When I hand in my damage claims according to the rules of Chom-Insurance, I do so because the insurance company often carries out controls.”), and voluntary cooperation (three items; e.g., “When I hand in my damage claims according to the rules with Chom-Insurance, I do so because the insurance company supports me if I have unintentionally filled in my damage claim incorrectly.”). Analog to the tax context, the participants' perceptions regarding wielded coercive power (four items; e.g., “I believe that the insurance company Chom-Insurance persecutes insurance fraudsters with audits and fines.”) and legitimate power (22 items; e.g., “I believe that the insurance company Chom-Insurance knows how to give good advice to insurants.”) of the insurance organization were adapted for insurance and assessed as manipulation check. Responses were indicated on a seven-point Likert scale ranging from 1 (“*I totally disagree*”) to 7 (“*I totally agree*”). Cronbach's α's are excellent and presented in Table 7. Socio-demographics (gender, age, income, nationality, employment, and experience with insurance organizations) were also assessed.

**Table 7:** **Study 4: Results of the ANOVAs with *coercive power* and *legitimate power* as independent variables**.

**Dependent variables**		**α**	***F* (df_1_, df_2_)**	***p***	**η_*p*_^2^**
Perceptions of coercive power		0.87			
	CP		69.94 (1, 98)	<0.001	0.42
	LP		0.90 (1, 98)	0.35	<0.01
	CPxLP		5.99 (1, 98)	0.02	0.06
Perceptions of legitimate power		0.94			
	CP		0.46 (1, 98)	0.50	<0.01
	LP		16.80 (1, 98)	<0.001	0.15
	CPxLP		0.16 (1, 98)	0.69	<0.01
Implicit trust		0.95			
	CP		1.14 (1, 98)	0.29	0.01
	LP		1.43 (1, 98)	0.23	0.01
	CPxLP		0.00 (1, 98)	0.29	0.01
Reason-based Trust		0.88			
	CP		0.01 (1, 98)	0.94	<0.01
	LP		23.67 (1, 98)	<0.001	0.20
	CPxLP		0.00 (1, 98)	0.95	<0.01
Antagonistic climate		0.89			
	CP		7.62 (1, 98)	<0.01	0.07
	LP		4.47 (1, 98)	0.04	0.04
	CPxLP		1.73 (1, 98)	0.19	0.02
Service climate		0.88			
	CP		1.08 (1, 98)	0.30	0.01
	LP		26.69 (1, 98)	<0.001	0.21
	CPxLP		0.30 (1, 98)	0.59	<0.01
Enforced compliance		0.89			
	CP		4.56 (1, 98)	0.04	0.04
	LP		3.30 (1, 98)	0.07	0.03
	CPxLP		0.23 (1, 98)	0.63	<0.01
Voluntary cooperation		0.81			
	CP		2.73 (1, 98)	0.13	0.02
	LP		10.72 (1, 98)	0.001	0.10
	CPxLP		0.06 (1, 98)	0.81	<0.01
Intended insurance fraud					
	CP		2.03 (1, 98)	0.16	0.02
	LP		0.00 (1, 98)	0.99	<0.01
	CPxLP		1.46 (1, 98)	0.23	0.02

*α, Cronbach α; CP, main effect coercive power; LP, main effect legitimate power; CPxLP, interaction effect of coercive and legitimate power*.

### Results

In the following, only hypothesized and/or significant results are reported; for completeness, Table 7 displays all findings independently, whether significant or not.

#### Preliminary data analyses

The manipulation check showed that perceptions on coercive power and legitimate power were induced in line with the manipulation (Table 7). Participants held perceptions of lower coercive power in the low levels of coercive power condition (cp_low_: *M* = 3.17, *SD* = 1.32) than in the high levels of coercive power condition (cp_high_: *M* = 5.28, *SD* = 1.31). A weak interaction of coercive and legitimate power on the perceptions of coercive power existed, but as this interaction explains only 6% of the variance and the main effect of coercive power explains 42%, this interaction is negligible. Participants experiencing high levels of legitimate power held perceptions of higher legitimate power (lp_high_: *M* = 4.74, *SD* = 1.14) than the participants experiencing low legitimate power (lp_low_: *M* = 3.79, *SD* = 1.15; Table 7).

#### Coercive power and legitimate power

##### The impact of coercive and legitimate power on trust, climates, and motives

Testing Hypotheses 1b and 2b, 2 (low vs. high levels of coercive power) by 2 (low vs. high levels of legitimate power) ANOVAs with the dependent variables implicit and reason-based trust, antagonistic and service climate and enforced compliance and voluntary cooperation were conducted. Similar to the experiments in the tax context, participants in conditions with low/high levels of coercive power and low/high levels of legitimate power reported equal intensity of *implicit trust* (main effects: cp_low_: *M* = 2.02, *SD* = 1.62; cp_high_: *M* = 2.35, *SD* = 1.55; lp_low_: *M* = 2.00, *SD* = 1.43; lp_high_ *M* = 2.40, *SD* = 1.70, see Table 7; for a graphical representation see Figure 1 in the Discussion Section). As expected, participants reported high levels of *reason-based trust* when legitimate power was high (lp_high_: *M* = 4.46, *SD* = 1.14; lp_low_: *M* = 3.22, *SD* = 1.37, see Table 7).

The analysis showed that, as expected, the perception of an *antagonistic climate* increased with coercive power (cp_low_: *M* = 2.84, *SD* = 1.71; cp_high_: *M* = 3.84, *SD* = 1.85) and decreased with legitimate power (lp_low_: *M* = 3.84, *SD* = 1.81; lp_high_: *M* = 2.99, *SD* = 1.81, see Table 7). Furthermore, as expected, the perception of a *service climate* increased only with legitimate power (lp_low_: *M* = 3.05, *SD* = 1.62; lp_high_: *M* = 4.58, *SD* = 1.33, see Table 7).

Regarding motives for cooperation, the analysis showed that, as expected, participants in conditions with low levels of coercive power reported feeling less *enforced* (cp_low_: *M* = 3.69, *SD* = 1.81) compared to conditions with high levels of coercive power (cp_high_: *M* = 4.37, *SD* = 1.66; see Table 7). As expected, participants reported more *voluntary cooperation* when legitimate power was high (lp_high_: *M* = 4.35, *SD* = 1.49; lp_low_: *M* = 3.37, *SD* = 1.44, see Table 7). No other main effects or interaction effects were significant (see Table 7).

##### The mediating role of trust, climate, and motive

Testing Hypothesis 3, an ANOVA revealed no significant main effect of coercive power, no impact of legitimate power and no significant interaction (Table 7).

In a second step, we applied again the program Mediate (Hayes et al., 2011) for the mediator analysis. The results revealed that there are three indirect effects from power to insurance fraud. First, legitimate power impacts insurance fraud intention via reason-based trust (95% CI [−20.80; −0.44], Table 8). Second, coercive power impacts insurance fraud intention via enforced compliance (95% CI [0.77; 10.64]) and third, legitimate power also impacts insurance fraud intention via enforced compliance (95% CI [0.28; 9.38]). However, Sobel test statistics revealed only a trend for a mediation from coercive power to enforced compliance to insurance fraud (CP→EC→IFI: Sobel test = −1.72, *p* =.08; *R*_M_ = −0.26). All other mediation were not significant (LP→RBT→IFI: Sobel test = −1.60, *p* = 0.11; LP→EC→IFI: Sobel test = 1.52, *p* = 0.13). The other indirect effects from coercive and legitimate power are not significant.

**Table 8:** **Study 4: Mediation analysis from power to insurance fraud intention (IFI; standard errors in parentheses)**.

	**Path coefficients**							**Indirect effects**		
	**To IFI**	**To IT**	**To RBT**	**To AC**	**To SC**	**To EC**	**To VC**	**Estimate**	**Sobel Z**	**Symmetric 95% CI**
From covercive power (CP)	−18.96 (7.39)	0.36 (0.32)	−0.02 (0.25)	0.94 (0.35)	−0.32 (0.29)	0.72 (0.34)	−0.45 (0.29)			
From legitimate power (LP)	1.83 (7.86)	0.42 (0.31)	1.24 (0.25)	−0.80 (0.35)	1.51 (0.29)	0.60 (0.34)	0.95 (0.29)			
From implicit trust (IT)	4.10 (2.43)									
From reason-based trust (RBT)	−7.99 (4.74)									
From antagonistic climate (AC)	2.23 (2.78)									
From service climate (SC)	1.45 (4.46)									
From enforced compliance (EC)	6.89 (2.32)									
From voluntary cooperation (VC)	0.76 (3.19)									
CP→IT→IFI								1.46 (1.75)	0.936	−0.69; 4.77
LP→IT→IFI								1.72 (1.81)	1.056	−0.51; 5.13
CP→RBT→IFI								0.15 (2.38)	0.080	−3.70; 4.21
LP→RBT→IFI								−9.89 (6.14)	−1.596	−20.80; −0.44
CP→AC→IFI								2.10 (2.89)	0.769	−2.09; 7.34
LP→AC→IFI								−1.77 (2.51)	−0.076	−6.31; 1.79
CP→SC→IFI								−0.46 (1.98)	−0.311	−4.08; 2.31
LP→SC→IFI								2.19 (6.89)	0.032	−8.62; 13.70
CP→EC→IFI								4.99 (3.09)	1.724	0.77; 10.64
LP→EC→IFI								4.16 (2.85)	1.517	0.28; 9.38
CP→VC→IFI								−0.35 (1.74)	−1.375	−3.43; 2.39
LP→VC→IFI								0.73 (3.15)	0.238	−4.29; 6.02

### Discussion

The predictions of Hypotheses 1b and 2b are partly confirmed. The combination of coercive and legitimate power backs up the prediction that coercive power impacts the antagonistic climate and enforced compliance (Hypothesis 1b). Furthermore, legitimate power had a positive impact on reason-based trust, the perception of a service climate and voluntary cooperation (Hypothesis 2b). In line with Study 3 but contrary to predictions, legitimate power reduced the antagonistic climate. In contrast to Studies 1–3, results of the mediator analysis showed that coercive power increases enforced compliance, which in turn decreases the intention to commit insurance fraud.

## General discussion

Overall, all four studies confirm the hypothesized impact of coercive and legitimate on cognitions when deciding to cooperate with authorities (Figure 1). As expected, when coercive power was applied exclusively, it decreased implicit trust, increased the perception of an antagonistic climate, and enforced compliance. For the combined prevalence of coercive and legitimate power, coercive power does not impact implicit trust, but leads to a perceived antagonistic climate and to an enforced motive to comply. The missing impact of coercive power on implicit trust, when combined with legitimate power, might stem from the fact that legitimate power stimulates rational considerations because of reason-based trust, and rational considerations are aspect of system 2, so that implicit trust (system 1) cannot arise (cf. Sittenthaler et al., 2015). Coercive power has a direct impact on tax cooperation intention and an indirect effect mediated via enforced compliance on insurance fraud intention.

As expected, legitimate power (wielded exclusively or in combination with coercive power) increases reason-based trust, the perception of a service climate and the motive to cooperate voluntarily. The relationship of legitimate power and intended cooperative behavior is mediated by reason-based trust.

Two unexpected results were found. First, in Study 2, and by tendency in Studies 3 and 4, legitimate power, contrary to expectations, increased the enforced motive to cooperate. One explanation is that due to feelings of reciprocity, even the wielding of legitimate power might make participants experience some “social” coercion that is responsible for motives of enforced compliance. This is in line with Ouchi's (1979) informal clan control, which sees reciprocity and a legitimate organization as the foundation. Additionally, social agreement, such as common values and beliefs, would constitute a further pre-requisite for clan control. Another possible reason is that legitimate power leads to the impression that authorities have a high proficiency for detecting and punishing defecting individuals, which results in feelings of enforced compliance. This result shows that a relations between legitimate power and enforced compliance needs to be included in the Slippery Slope Framework (Gangl et al., 2015) so that future research will consider this issue.

Second, contrary to expectations but in line with earlier findings (Hofmann et al., 2014), legitimate power, even when combined with coercive power, reduced the perceived antagonistic climate (Studies 2–4). When combined, the exertion of audits and fines (i.e., coercive power) can be believed to be legitimate and, thus, be accepted as the right thing to do. This assumption was supported by Study 2, which showed that coercive power is more pronounced when legitimate power is rather high (in this study, only legitimate power was manipulated and no information on coercive power was given). Then, trust in authorities and relational climates were more effected by legitimate power than by coercive power alone. This is suggested by the relatively strong impact of legitimate power on reason-based trust in Study 2–4. Overall, the present results certainly indicate a connection between coercive power and legitimate power. With the current experiments, this connection, e.g., how the application of legitimate power impacts the perception of coercive power, cannot sufficiently be tested, but the Slippery Slope Framework (Gangl et al., 2015) needs to be modified including a connection between coercive power and legitimate power and future research will have to investigate this aspect.

The current studies have some limitations that have to be addressed in future research. The research theoretically bases on the slippery slope framework postulating that authorities' different forms of power influence cognitions and subsequently cooperative behavior. It can be argued that this causal relationship could be the other way round that not power impacts cognitions but actual cognitions are responsible for the perceptions of power. This certainly can be the case and needs further empirical evidence, nevertheless, as our studies show, there certainly is a significant impact of power on cognitions. As with most laboratory experiments, the investigated samples are not representative, they are specifically comprised of students who are not well-experienced with tax authorities and/or insurance organizations. This, nonetheless, is actually an advantage. For naïve participants, it is easier to imagine the fictitious scenario and act based on the presented scenarios and not on prior experiences with the authority (Mittone, 2006). That said, laboratory experiments still create a highly artificial situation in which individuals might not behave as in an everyday context. Therefore, allowing participants to take part in an online experiment at home (Study 4) is a possibility to counteract this artificiality without changing manipulation. Nevertheless, future field experiments that not only investigate the direct impact of power on cooperation (e.g., Ariel, 2012; Gangl et al., 2014) but also investigate the underlying processes could strengthen the current results; tax authorities and/or insurance organizations displaying coercive and/or legitimate power would show the effects of power *in vivo*. Furthermore, the experimental design of the current study can only test for differences. The correlative connections between power and processes are only assumed. Thus, this design only allows for limited conclusions regarding the mediators since they are based on manipulated factors of fictitious authorities and not on actual existing authorities. However, due to the experimental setting, we were able to obtain high internal validity. Future research needs to increase external validity and address the studied relationships by using field data.

Literature indicates that the severity of punishment is contingent on the type of social dilemma situation (Molenmaker et al., 2014). It has to be mentioned that legal circumstances of tax authorities and insurance organizations are different. While in comparison to tax authorities, insurance organizations do not have the legal right to punish insurance fraud. Taxpayers, compared to insurance holders, also do not have the option to turn to another tax authority if they are not satisfied with a specific tax authority's conduct. Taxpayers are at the mercy of one specific tax authority in a certain country. Nevertheless, results on the impact of power work similarly in both contexts. The two authorities in the studies, the tax authority and the insurance organization, represent a small range of authorities that wield power to control individuals' behavior in different situations. In future research, other institutions, such as governments ensuring citizens' environmental friendliness, should be investigated. Research on how their power affects trust, relational climates, and motives for cooperation will further support, as well as extend, current findings.

From a practical point of view, the present findings are of value, not only for tax authorities and insurance companies, but for all authorities wielding power. Results show that sanctions of undesired behavior, as well as legitimate procedures, both not only foster cooperation, but also have different impacts on underlying cognitions. Severe punishments lead to a hostile and antagonistic climate that should be avoided, whereas supportive procedures foster trust toward the authority and the perception of a reciprocative service climate. Legitimate power operates via establishing reason-based trust. Tax authorities and insurance organizations are supposed to reduce costly punishments, provide supportive procedures and helpful information, and pursue societal goals to assure a service climate. This would, in the long run, create trust toward them which fosters cooperative behavior. The findings also indicate that strict audits and severe fines might alienate individuals that are either reacting with enforced compliance or looking for more appealing alternatives. Thus, the current results should initiate rethinking power of all authorities shaping individual behavior.

Highlighting the mechanisms by experimentally showing how coercive and/or legitimate power of authorities affect trust in authorities, the relational climate, and their motives over different contexts expanded the understanding of the operating mode of authorities' power. While the mediating effects clearly show that a key factor in understanding the mechanisms is reason-based trust, implicit trust, the relational climates and motives to comply become of marginal interest. They are mainly a product of specific forms of power, but they do not interfere with the actual connection of power and behavioral intention. These findings have extensive consequences for theory, as well as for real world authorities, giving direction for future research and specifying actions for power wielding authorities. In a nutshell, trust building measures are central, as reason-based trust is mediating the impact of power on cooperation, but other cognitions (interaction climates, motives) might not have that importance for cooperation.

## Ethics statement

The present study was conducted in accordance with the Declaration of Helsinki (7th revision, 2013) and local ethical guidelines for experimentation with human participants at the Faculty of Psychology of the University of Vienna. All participants gave written informed consent prior to the experiment.

## Author contributions

EH: Research design, conduction of Studies 1–4, analyses of Studies 1–4, drafting of article. BH: Conduction of Study 4, analyses of Studies 1–4, drafting of article. KG: Research design, analyses of Studies 1–4, drafting of article. MH: Conduction of Studies 1–4, drafting of article. EK: Drafting of article.

## Funding

This research was financed by grant number P24863-G16 from the Austrian Science Fund (FWF) (http://www.fwf.ac.at/en/).

### Conflict of interest statement

The authors declare that the research was conducted in the absence of any commercial or financial relationships that could be construed as a potential conflict of interest.

## Footnotes

^1^Although, enforced compliance and voluntary cooperation seem similar to the concept of extrinsic and intrinsic motivation (Ryan and Deci, 2000), this is not the case. While enforced compliance is comparable to extrinsic motivation based on external regulation, voluntary cooperation can neither be classified as other forms of extrinsic (based on introjection, identification, integration) or intrinsic information, because it is actually a reciprocal act.

^2^All ANOVAs (studies 1–4) were also undertaken as ANCOVAs with socio-demographic control variables, resulting in the same results as the reported ANOVA results.

^3^We us *R*_M_, the ration of the indirect effect to the direct effect, as an effect size for the mediation (Preacher and Kelley, 2011).
