# Supplementary material for: Authorities' Coercive and Legitimate Power: The Impact on Cognitions Underlying Cooperation
Source: Front Psychol. 2017 Jan 18;8:5. doi: 10.3389/fpsyg.2017.00005 (PMC5241301; doi:10.3389/fpsyg.2017.00005)
Supplement: Supplementary file 1 [file DataSheet1.docx]

**Appendix**

Items are translated from the original German into English.

**Study 1-3**

| **Coercive power** |
| --- |
| *I believe that the tax authority …* |
| severely punishes tax evaders. |
| enforces its demands through audits and penalties. |
| punishes severely, when it finds a mistake. |
| persecutes taxpayers with audits and fines. |
| **Legitimate power** |
| *I believe that the tax authority …* |
| depends on taxpayers who fill out their tax forms correctly in order to work efficiently. |
| knows, how to give good advice to taxpayers. |
| provides taxpayers with professional advice. |
| should receive a diligently completed tax return if past tax returns were often carelessly or erroneously completed. |
| arranges comprehensible procedures for the collection of taxes. |
| is an institution taxpayers feel obliged to cooperate with because it has rendered them many services in the past, e.g., child support. |
| holds counseling sessions, at which the correct way to file tax returns is demonstrated in minute detail so that taxpayers can learn. |
| is an institution that taxpayers feel obliged to because it has accommodated them in past audits. |
| ensures that concerns of taxpayers are processed efficiently and fast. |
| can only provide good advice and information if taxpayers are honest. |
| deserves an honest tax return if tax obligations have often not been met in the past. |
| has the right to persecute tax evasion. |
| should receive correct tax returns if previous tax returns were completed very carelessly. |
| needs taxpayers’ cooperation to fulfill its duties properly. |
| is appreciated by taxpayers for providing comprehensive information and advice. |
| has the legal obligation to collect taxes. |
| is valued by taxpayers for the high standards it sets itself regarding the collection of taxes. |
| is valued by taxpayers for using audits additionally to advise taxpayers. |
| is legally obligated to advise taxpayers on tax-related matters. |
| supplies comprehensive information that helps taxpayers orient themselves as to how to pay their taxes correctly. |
| is an institution taxpayers feel obliged to because it has considered their concerns in the past. |
| occupies employees, who inform comprehensively about tax issues. |
| **Implicit trust** |
| *I trust the tax authority in Chomland …* |
| without thinking about it. |
| blind. |
| automatically. |
| **Reason-based trust** |
| *I trust the tax authority in Chomland …* |
| because it has the necessary political support for its work. |
| because I depend on it. |
| because it occupies motivated employees who give comprehensive advice. |
| because it means no financial risk for me. |
| because it gives me competent advice. |
| because it pursues the important goal, to offer competent advice. |
| because it benevolently advises the taxpayers regarding tax issues. |
| **Antagonistic climate** |
| Between the tax authority in Chomland and taxpayers there exists a climate … |
| of inconsiderateness. |
| of ruthlessness. |
| of ‘cops and robbers‘. |
| **Service climate** |
| Between the tax authority in Chomland and taxpayers there exists a climate … |
| in which taxpayers are customers of the tax authorities. |
| like between an enterprise and their customers. |
| which is characterized by its service-oriented nature. |
| **Enforced compliance** |
| When I pay taxes according to the law in Chomland, I do so … |
| because a great many tax audits are carried out. |
| because I know I will be audited. |
| because the tax authority often carries out audits. |
| **Voluntary cooperation** |
| When I pay taxes according to the law in Chomland, I do so … |
| because the tax authority will probably reciprocate my cooperation. |
| because the tax authority treats me correctly as long as I admit mistakes. |
| because the tax authority supports taxpayers who make unintentional mistakes. |

**Study 4**

| **Coercive power** |
| --- |
| *I believe that the insurance company Chom-Insurance …* |
| severely punishes insurance fraudsters. |
| enforces its demands through audits and penalties. |
| punishes severely, when it finds a mistake. |
| persecutes taxpayers with audits and fines. |
| **Legitimate power** |
| *I believe that the insurance company Chom-Insurance …* |
| depends on insurants who fill out their insurance forms correctly in order to work efficiently. |
| knows, how to give good advice to insurants. |
| provides insurants with professional advice. |
| should receive a diligently completed insurance form if past forms were often carelessly or erroneously completed. |
| arranges comprehensible procedures for the settlement of claims. |
| is an institution insurants feel obliged to cooperate with because it has rendered them many insurance benefits in the past (e.g., with damages of insured property, etc.). |
| holds counseling sessions, at which the correct way to fill in insurance forms is demonstrated in minute detail so that taxpayers can learn. |
| is an institution that insurants feel obliged to because it has rendered them good services in the past. |
| ensures that concerns of insurants are processed efficiently and fast. |
| can only provide good advice and information if insurants are honest. |
| deserves an honest insurance forms if incorrect damages have often been reported in the past. |
| has the right to persecute insurance fraud. |
| should receive correct insurance forms if previous insurance forms were completed very carelessly. |
| needs insurants’ cooperation to fulfill its duties properly. |
| is appreciated by insurants for providing comprehensive information and advice. |
| has the legal obligation to prosecute insurance fraud. |
| is valued by insurants for the high standards it sets itself regarding the prosecution of insurance fraud. |
| is valued by taxpayers for using controls additionally to advise insurants. |
| is legally obligated to advise insurants on insurance-related matters. |
| supplies comprehensive information that helps insurants orient themselves as to how to hand in their insurance claims. |
| is an institution insurants feel obliged to because it has considered their concerns in the past. |
| occupies employees, who inform comprehensively about insurance issues. |
| **Implicit trust** |
| *I trust the insurance company Chom-Insurance …* |
| without thinking about it. |
| blind. |
| automatically. |
| **Reason-based trust** |
| *I trust the insurance company Chom-Insurance …* |
| because it hast to act upon the law which protects the interests of insurants. |
| because I depend on it. |
| because it occupies motivated employees who give comprehensive advice. |
| because it means no financial risk for me. |
| because it gives me competent advice. |
| because it pursues the important goal, to offer competent advice. |
| because it benevolently advises the insurants regarding tax issues. |
| **Antagonistic climate** |
| Between the insurance company Chom-Insurance and the insurants there exists a climate … |
| of inconsiderateness. |
| of ruthlessness. |
| of ‘cops and robbers‘. |
| **Service climate** |
| Between the insurance company Chom-Insurance and the insurants there exists a climate … |
| which is characterized by its service-oriented nature. |
| in which insurants are treated like valued customers of the insurance company. |
| like between an enterprise and their longtime customers. |
| **Enforced compliance** |
| When I hand in my damage claims according to the rules with Chom-Insurance, I do so … |
| because a great many controls are carried out. |
| because I know I will be controlled. |
| because the insurance company often carries out controls. |
| **Voluntary cooperation** |
| When I hand in my damage claims according to the rules with Chom-Insurance, I do so … |
| because the insurance company will probably reciprocate my cooperation. |
| because the insurance company treats me correctly as long as I admit mistakes. |
| because the insurance company supports me, if I have unintentionally filled in my damage claim incorrectly. |
